# Microwave-Assisted Syntheses in Recyclable Ionic Liquids: Photoresists Based on Renewable Resources

**DOI:** 10.1002/cssc.201500847

**Published:** 2015-09-10

**Authors:** Charlotte Petit, Klaus P Luef, Matthias Edler, Thomas Griesser, Jennifer M Kremsner, Alexander Stadler, Bruno Grassl, Stéphanie Reynaud, Frank Wiesbrock

**Affiliations:** [a] IPREM, UMR 5254 UPPA/CNRS, Hélioparc2 Avenue du Président Angot, 64053, Pau CEDEX 09 (France) E-mail : stephanie.reynaud@univ-pau.fr; [b] Polymer Competence Center Leoben (PCCL)Roseggerstrasse 12, 8700, Leoben (Austria); [c] Institute for Chemistry and Technology of Materials, Graz University of Technology, NAWI GrazStremayrgasse 9, 8010, Graz (Austria); [d] Chair of Chemistry of Polymeric Materials, University of LeobenOtto-Gloeckel-Strasse 2, 8700, Leoben (Austria); [e] Anton Paar GmbH (Ltd.)Anton-Paar-Strasse 20, 8054, Graz (Austria)

**Keywords:** copolymerization, ionic liquids, microwave chemistry, renewable resources, ring-opening polymerization

## Abstract

The copoly(2-oxazoline) pNonOx_80_-*stat*-pDc^=^Ox_20_ can be synthesized from the cationic ring-opening copolymerization of 2-nonyl-2-oxazoline NonOx and 2-dec-9′-enyl-2-oxazoline Dc^=^Ox in the ionic liquid *n*-hexyl methylimidazolium tetrafluoroborate under microwave irradiation in 250 g/batch quantities. The polymer precipitates upon cooling, enabling easy recovery of the polymer and the ionic liquid. Both monomers can be obtained from fatty acids from renewable resources. pNonOx_80_-*stat*-pDc^=^Ox_20_ can be used as polymer in a photoresist (resolution of 1 μm) based on UV-induced thiol–ene reactions.

Photolithography is a standard procedure for the production of 2.5-dimensional polymer structures.[1–3] A polymer film is illuminated through a mask, subjecting the illuminated areas of the photoresist film to photochemical reactions that change the solubility of the polymer. In the case of so-called negative photoresists, the illuminated areas become insoluble while the non-illuminated areas stay soluble and, consequently, can be dissolved in a subsequent development step, yielding a structured polymer film that reproduces the geometric pattern preset by the mask in negative fashion.

In earlier studies, we reported negative photoresists based on copoly(2-oxazoline)s that can be crosslinked upon illumination with UV light.[4,5] As crosslinking reaction, the thiol–ene reaction, a prominent congener of the click reactions, was chosen.[6,7] Copoly(2-oxazoline)s bearing unsaturated double bonds in their side chains can be crosslinked with bisfunctional thiols, such as butane dithiol and 3,4-dimercapto toluene, or tetrafunctional thiols, such as pentaerythritol-tetra-(3-mercaptoproprionate), yielding structured polymer films with a resolution of 2 μm.

In order to further advance this toolbox of copoly(2-oxazoline)-based photoresists, we have redesigned our synthetic strategy meeting the current demand for more environmentally benign syntheses, aiming at the elimination of volatile organic compounds as reaction medium [i.e., using ionic liquids (ILs) as substitute][8–11] and the employment of reactants from renewable resources (i.e., fatty acids as resource).

Polymerizations in ILs have previously focused on radical polymerizations as prominent examples.[12] In the area of poly(2-oxazoline)s, the polymerization of 2-ethyl-2-oxazoline in ILs has been investigated in a detailed kinetic study.[13] Notably, for the polymerization of 2-oxazolines, which are commonly performed under microwave irradiation nowadays,[14–16] ILs offer the additional advantage that, due to their ionic character, they are prime absorbers of microwave irradiation, which paves the way for a highly energy-efficient process.[17]

In this study, the polymerization of 2-oxazolines in ILs is expanded to the preparation of functional materials. Based on earlier studies on the application of copoly(2-oxazoline)s as photoresist,[4,5] two different 2-oxazoline monomers for the synthesis of the copoly(2-oxazoline) should be chosen: one with an unsaturated double bond in its side chain and another with a nonfunctionalized side chain. In order to meet the criteria for green synthesis as comprehensively as possible, several considerations were taken into account. Among them, organic solvents should not be involved in the monomer syntheses. This prerequisite eliminated double-bond-bearing 2-oxazoline monomers with short side chains such as 2-but-3′-enyl-2-oxazoline, the synthesis of which requires vast amounts of halogenated solvents.[18] 2-Oxazoline monomers with longer side chains, on the other hand, can be synthesized from the reaction of ethanol amine with either the corresponding nitriles[19] or the corresponding carboxylates or carboxylic acids.[20] As the reaction involving nitriles requires catalysis by toxic cadmium compounds, the reaction involving carboxylates or carboxylic acids (which is catalyzed by titanium compounds) was favored.

Finally, the two monomers for the synthesis of the copoly(2-oxazoline), namely 2-nonyl-2-oxazoline (NonOx) and 2-dec-9′-enyl-2-oxazoline (Dc^=^Ox), were synthesized from the reaction of decanoic acid (available from renewable resources such as coconut oil)[21] and undec-10-enoic acid (available from renewable resources such as castor oil),[22] respectively, with ethanol amine (Scheme 1). Ethanol amine, which is commonly synthesized by the reaction of ethylene oxide and ammonia, is produced in animal tissue as the biogenic amine of serine as well.[23] The condensation reactions were catalyzed by titanium alcoholates and could be performed without the addition of any solvents, with moderate yields of approx. 60 % for the purified monomers after distillation from the reaction mixture. The copolymerization of the monomers was performed as cationic ring-opening polymerization,[14,15] initiated by methyl tosylate (Scheme 2) in an IL under microwave irradiation. A ratio NonOx/Dc^=^Ox=80:20 was chosen based on previous findings for poly(2-oxazoline)-based photoresists.[4]

**Scheme 1:**
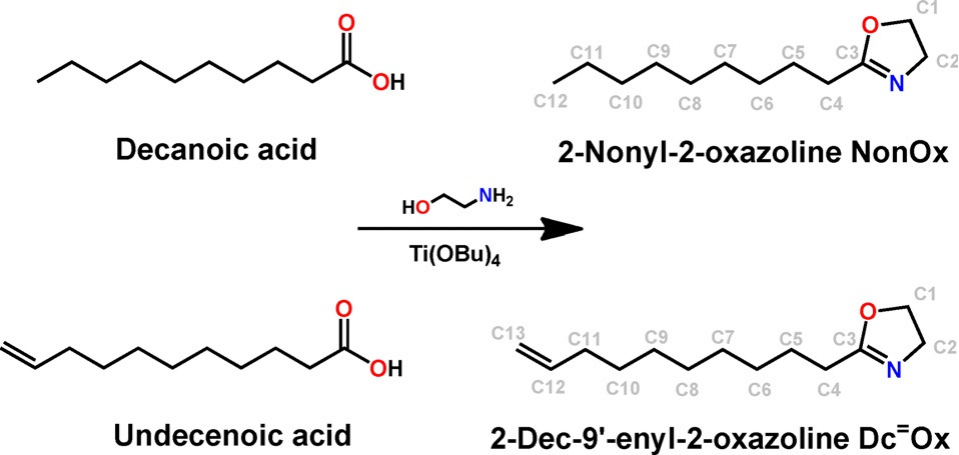
Representation of the synthesis of the two 2-oxazoline monomers (with atom numbering) from carboxylic acids derived from renewable resources.

**Scheme 2:**
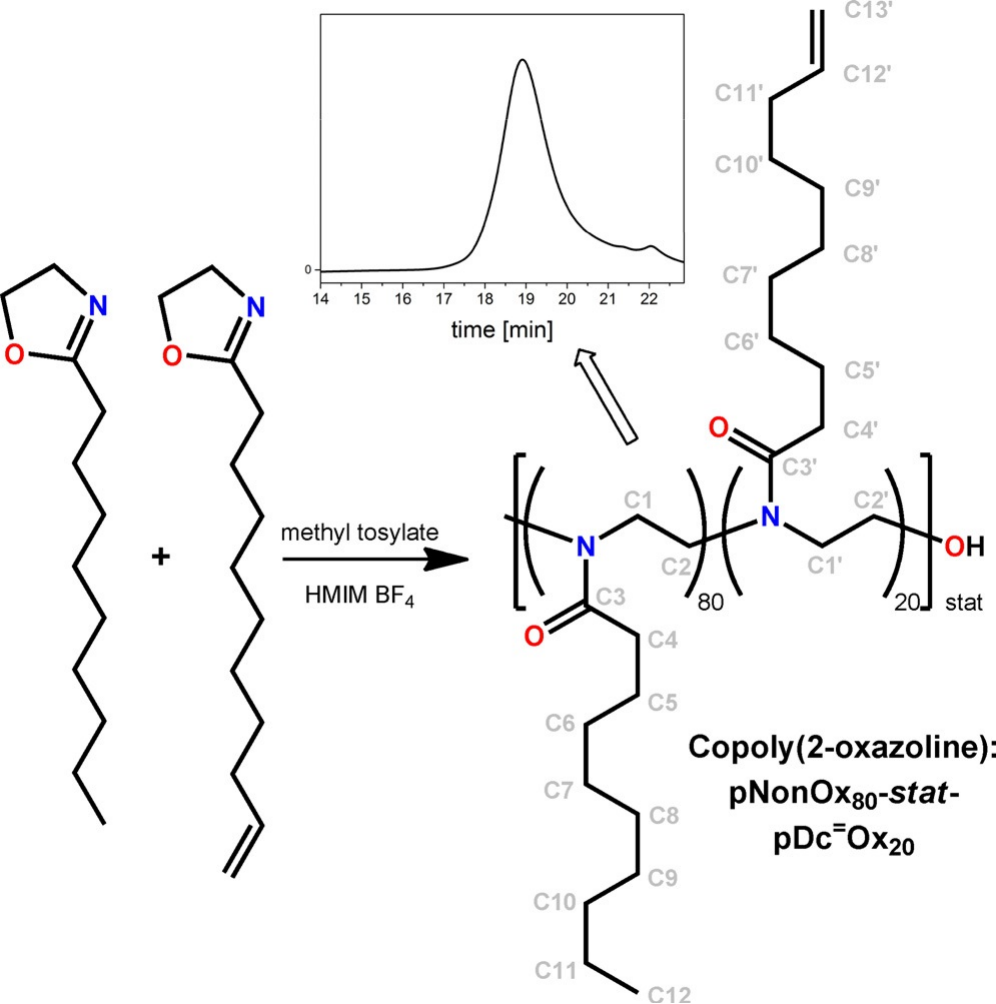
Representation of the cationic ring-opening copolymerization of NonOx and Dc^=^Ox, yielding the statistical copolymer pNonOx_80_-*stat*-pDc^=^Ox_20_ (with atom numbering). The size exclusion chromatogram (details of the SEC: see Supporting Information) of the large-scale synthesis (250 g of polymer) is shown as an insert.

*n*-Hexyl methylimidazolium tetrafluoroborate (HMIM BF_4_) was chosen as reaction medium because of its solubility properties: While it dissolves the monomers and the initiator and is miscible with the molten copoly(2-oxazoline), it does not dissolve the product pNonOx_80_-*stat*-pDc^=^Ox_20_, which precipitates upon cooling of the reaction mixture.

The reaction parameters for the microwave-assisted polymerization were optimized in a Monowave 300 reactor with magnetic stirring (600 rpm) at a small scale (reaction mixtures of 10 g each). A reaction temperature of 140 °C and irradiation times of 90 min were found to be sufficient for complete monomer conversion. The large-scale polymerizations (reaction mixtures of 750 g) were performed at these optimized parameters in a Masterwave BTR (bench top reactor), equipped with mechanical stirring. After the initial heating of the reaction mixture (lasting 5.5 min with an average power input of 935 W; maximum input power: 1700 W), an average power input of only 170 W was sufficient to maintain the targeted reaction temperature for the targeted polymerization time of 90 min.

The polymer precipitated spontaneously from the IL and could be recovered after cooling in crude quality by filtration. As some IL was occluded in the polymer, the product needed to be further purified by dispersion in distilled water and ultrasonification for 30 min. The polymer recovered by this second filtration did not show any impurities according to NMR analysis. The aqueous phase was combined with the IL from the first filtration. After thorough drying at reduced pressure and elevated temperatures, the IL did not show any impurities either and could be reused as reaction medium. Both, the copoly(2-oxazoline) as well as the IL, were recovered in quantities of 95 %.

The targeted composition of the copoly(2-oxazoline) (ratio NonOx/Dc^=^Ox=80:20) was confirmed by ^1^H NMR analyses. The weight dispersity of the polymer was determined by size exclusion chromatography to be 1.34 (calculated from *M_w_*=10.8 kDa and *M_n_*=8.1 kDa, according to the PS calibration) (Scheme 2), which is of a reasonable range for this synthetic scale (250 g of polymer/batch from 750 g reaction mixtures) and is assumed to originate in part from the efficient stirring provided.

As crosslinking method, the UV-induced thiol–ene reaction was chosen because of the high yields obtainable with this reaction. Hence, for the formulation of the photoresist, in addition to the ‘ene’ component [the copoly(2-oxazoline) pNonOx_80_-*stat*-pDc^=^Ox_20_], also an oligofunctional thiol component, a photoinitiator, and a solvent needed to be chosen. Because of its high reactivity in thiol–ene reactions, pentaerythritol-tetra-(3-mercaptoproprionate) (4SH) was chosen as thiol. Ethyl lactate proved to be a suitable green solvent for both reactants.[24] 2,4,6-Trimethylbenzoylethylphenylphosphinate (Irgacure TPO-L) was chosen as photoinitiator because of its high reactivity and good dispersability in ethyl lactate.

The photoresist formulations could be applied onto various substrates such as FR4, gold-coated FR4, glass, and calcium fluoride, by either drop- or spincasting. After removal of the residual solvent at elevated temperatures, smooth nonsticky surfaces were obtained. Hence, masks could be put on the polymer films during illumination in direct contact.

Due to the oligofunctionality of the thiol and the copoly(2-oxazoline) and a maintenance of the thiol/ene ratio of 1:1, efficient crosslinking could be achieved in UV illumination times as short as 1 min. According to sol–gel analyses of photoresists that were illuminated without a mask, the thiol–ene reaction proceeded to a degree that no soluble fractions could be detected; IR analyses of illuminated films failed to reveal the signal of the thiol group. The illuminated photoresists could be developed in ethyl lactate; best results were obtained if the ethyl lactate was applied at slightly elevated temperatures of 40 °C. The height profile of the polymer film after development (Figure 1) revealed comparably smooth surfaces (height changes of ±5 nm in a film with a height of 100 nm) and only weakly pronounced flank angles at its edges.

**Figure 1:**
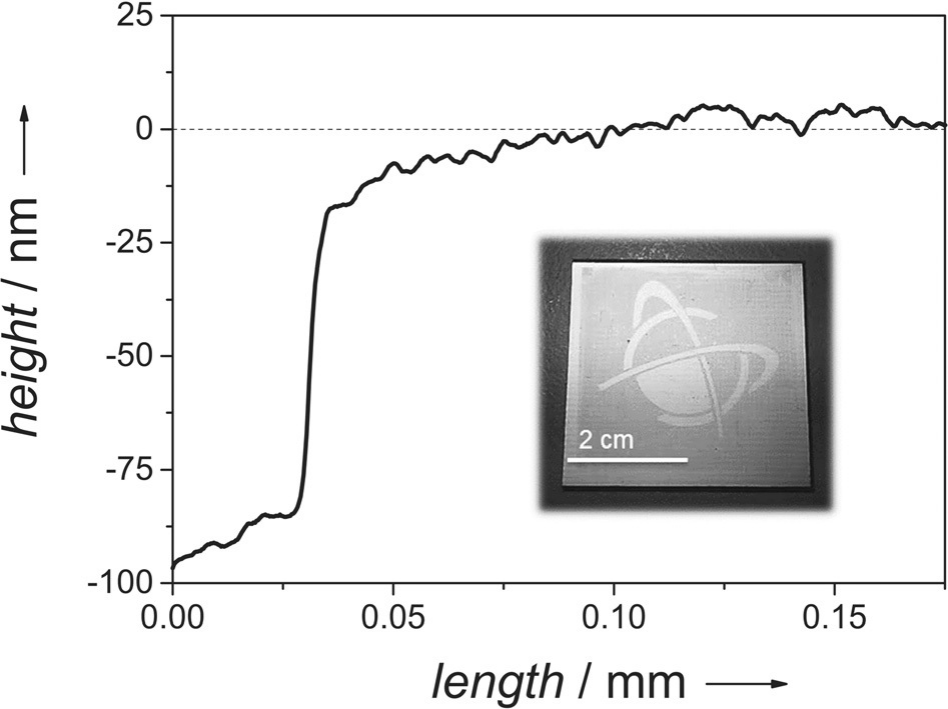
Height profile of a film of pNonOx_80_-*stat*-pDc^=^Ox_20_ after illumination and development. The copoly(2-oxazoline) had been spincast on a gold-coated FR4 substrate and been illuminated through a mask.

The resolution attainable with the pNonOx_80_-*stat*-pDc^=^Ox_20_-based photoresist was determined on the example of calcium fluoride substrates, onto which the photoresist formulation was spincast. After illumination through a mask aligner system equipped with a quartz-chromium mask, the polymer films were developed in ethyl lactate. Optical microscopy of the developed films (Figure 2) revealed resolutions as high as 1 μm, representing the highest resolution reachable and detectable with the set-up employed.[25]

**Figure 2:**
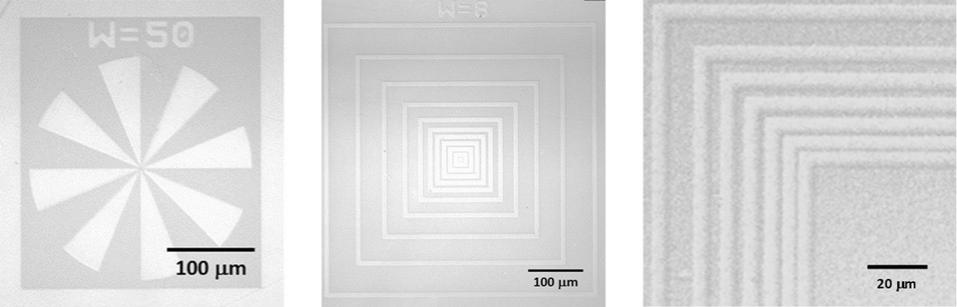
Light microscopy images of the developed photoresist.

The 2-oxazoline monomers NonOx and Dc^=^Ox were synthesized from renewable resources; only three types of solvents were required from the synthesis of the monomers to the readily-produced 2.5-dimensional polymer films: the IL HMIM BF_4_ as reaction medium for the cationic ring-opening polymerization of the 2-oxazolines (which can be recovered and re-used), water for the purification of the crude pNonOx_80_-*stat*-pDc^=^Ox_20_, and ethyl lactate for the formulation and development of the photoresist. Of special additional notice are the monomer syntheses from renewable resources and the low power consumption for the microwave-assisted polymerization and the straightforward recovery of the IL HMIM BF_4_ and the copolymer pNonOx_80_-*stat*-pDc^=^Ox_20_. Hence, in comparison with previously reported copoly(2-oxazoline)-based photoresists, the congener reported in here combines numerous approaches towards an environmentally more benign synthesis and application, while not showing any disadvantages in the final material: The 2.5-dimensional structures of the developed polymer film exhibit smooth surfaces and resolutions of 1 μm, and good adhesion to various substrates.

## Supporting Information

As a service to our authors and readers, this journal provides supporting information supplied by the authors. Such materials are peer reviewed and may be re-organized for online delivery, but are not copy-edited or typeset. Technical support issues arising from supporting information (other than missing files) should be addressed to the authors.

## References

[1] Marqués-Hueso J, Abargues R, Valdes JL, Martinez-Pastor JP (2010). J. Mater. Chem.

[2] Moon S-Y, Kim J-M (2007). J. Photochem. Photobiol. C.

[3] Staab M, Greiner F, Schlosser M, Schlaak HF (2011). J. Microelectromech. Syst.

[4] Schenk V, Ellmaier L, Rossegger E, Edler M, Griesser T, Weidinger G, Wiesbrock F (2012). Macromol. Rapid Commun.

[5] Fimberger M, Schenk V, Rossegger E, Wiesbrock F (2014). Period. Polytech. Chem. Eng.

[6] Hoyle CE, Bowman CN (2010). Angew. Chem. Int. Ed.

[b01] (2010). Angew. Chem.

[7] Kade MJ, Burke DJ, Hawker CJ (2010). J. Polym. Sci. Part A.

[8] Smiglak M, Pringle JM, Lu X, Han L, Zhang S, Gao H, MacFarlane DR, Rogers RD (2014). Chem. Commun.

[9] Torimoto T, Tsuda T, Okazaki K, Kuwabata S (2010). Adv. Mater.

[10] Lu J, Yan F, Texter J (2009). Prog. Polym. Sci.

[11] Egorova K, Ananikov VP (2014). ChemSusChem.

[12] Kubisa P (2004). Prog. Polym. Sci.

[13] Guerrero-Sanchez C, Hoogenboom R, Schubert US (2006). Chem. Commun.

[14] Wiesbrock F, Hoogenboom R, Abeln CH, Schubert US (2004). Macromol. Rapid Commun.

[15] Wiesbrock F, Hoogenboom R, Leenen MAM, Meier MAR, Schubert US (2005). Macromolecules.

[16] Ebner C, Bodner T, Stelzer F, Wiesbrock F (2011). Macromol. Rapid Commun.

[17] Kappe CO (2004). Angew. Chem. Int. Ed.

[b02] (2004). Angew. Chem.

[18] Gress A, Völkel A, Schlaad H (2007). Macromolecules.

[19] Witte H, Seeliger W (1974). Liebigs Ann. Chem.

[20] Krause H-J, Neumann P (1995).

[21] Mol JC (2002). Green Chem.

[22] Van der Steen M, Stevens CV (2009). ChemSusChem.

[23] Dennis EA, Kennedy EP (1972). J. Lipid Res.

[24] Pereira CSM, Silva VMTM, Rodrigues AE (2011). Green Chem.

[25] Jonkheijm P, Weinrich D, Köhn M, Engelkamp H, Christianen PCM, Kuhlmann J, Maan JC, Nüsse D, Schroeder H, Wacker R, Breinbauer R, Niemeyer CM, Waldmann H (2008). Angew. Chem. Int. Ed.

[b03] (2008). Angew. Chem.

