# Supplementary material for: Microwave-Assisted Syntheses in Recyclable Ionic Liquids: Photoresists Based on Renewable Resources
Source: ChemSusChem. 2015 Sep 10;8(20):3401–4. doi: 10.1002/cssc.201500847 (PMC4641455; doi:10.1002/cssc.201500847)
Supplement: Supplementary file 1 — Supplementary [file cssc0008-3401-sd1.pdf]

CHEMISTRY & SUSTAINABILITY
CHEMSUSCHEM
ENERGY & MATERIALS

Supporting Information

Microwave-Assisted Syntheses in Recyclable Ionic Liquids: Photoresists Based on Renewable Resources

Charlotte Petit,[a] Klaus P. Luef,[b, c] Matthias Edler,[d] Thomas Griesser,[d] Jennifer M. Kremsner,[e] Alexander Stadler,[e] Bruno Grassl,[a] Stéphanie Reynaud,*[a] and Frank Wiesbrock*[b]

cssc_201500847_sm_miscellaneous_information.pdf

Experimental Part

Materials and methods

All materials were purchased from Sigma-Aldrich (Vienna, Austria) except for the ionic liquid IL *n*-hexyl methylimidazolium tetrafluoroborate HMIM BF_4, which was delivered by Iolitec (Heilbronn, Germany), and the photoinitiator Lucirin TPO-L, which was delivered by abcr (Karlsruhe, Germany). Methyl tosylate was distilled prior to use; HMIM BF_4 was stored at reduced pressure at ambient pressure. All other chemicals were used as received.

^{1}H-NMR spectra were recorded on a Bruker Advance 300 MHz spectrometer with 64 scans and a relaxation delay of 4 s. The solvent residual peak of deuterated chloroform was used for referencing the spectra to 7.26 ppm.

Average molecular weights and dispersities were determined by size exclusion chromatography GPC using $CHCl_3/Et_3N/^{iso}PrOH$ (94/4/2) as eluent. The measurements were performed with a Merck Hitachi L-6000A pump, separation columns from Polymer Standard Service, 8\300 µm STV linear XL 5 µm-grade size, and a differential refractometer Waters 410 detector. For calibration, polystyrene standards from Polymer Standard Service were used.

Height profiles were measured on the Veeco Dektak 150. For cross hatch tests, the cutter 295/IX of Erichsen was used.

IR spectra were recorded an a Bruker Alpha FT-IR spectrometer equipped with ALPHA's Platinum ATR single reflection diamond ATR module.

The photoresist formulations were spincast on the spincoater SUSS CT-62 operated at 2500 rpm.

Photo patterning was performed with Hg/Xe lamps of the EFOS Novacure from EXFO for 60 s (6500 mW/cm^2, 7.5 cm distance lamp-substrate) or on the mask aligner system MJB4 from SUSS with a quartz-chromium mask at 500 mW for 60 s in contact mode, respectively.

Synthetic procedures

Small-scale syntheses (10 mL) were performed in the Anton Paar microwave reactor Monowave 300. The temperature was measured with an external IR pyrometer. The polymerizations were performed at 140 °C during 90 min in capped G30 vials, which contained 10 mL of reaction solution. Stirring was provided by a magnetic stirrer (600 rpm). The microwave reactor heated up the reaction mixture as fast as possible with a power maximum set to 150 W. At the end of the reaction, the vials were cooled to 55 °C by compressed air prior to the release of the vial from the reactor. Large-scale syntheses (700 mL) were performed in the Anton Paar Masterwave BTR microwave reactor equipped with an internal Pt100 temperature sensor. Mechanical stirring was performed at 600 rpm. The targeted

reaction temperature of 140 °C was reached within 5.5 min; approx. 170 W were required to maintain the reaction temperature. The reaction was run for 90 min; subsequently, the reactor was cooled to 70 °C.

Synthesis of the 2-oxazoline monomers

2-Nonyl-2-oxazoline NonOx was synthesized from 312.5 g (1.814 mol; 1 eqv.) of decanoic acid, 166 g (2,718 mol; 1.5 eqv.) of ethanolamine and 6.25 g of titanium(IV) butoxide (0.018 mmol), which were added without any additional solvent in a flask and were stirred under reflux at 160 °C for 24 h. Additional equivalents of titanium(IV) butoxide of 6.25 g each were added after 16 and 20 h, respectively. After removal of the reflux cooling unit, the solution was stirred for another 24 h at 160 °C. Subsequently, the NonOx monomer was recovered by distillation at 175 °C at reduced pressure (0.2 mbar). 230 g (1.166 mol) of purified product were obtained; yield: 64%.

^{1}H-NMR (300 MHz, $CDCl_3$); for atom numbering, see Scheme 1: δ (ppm) = 4.21 (2 H, t, $^3J_{H,H}$ = 9.4 Hz, H2), 3.81 (2 H, t, $^3J_{H,H}$ = 9.4 Hz, H1), 2.25 (2 H, m, H4), 1.58-1.66 (2 H, m, H11), 1.25 (12 H, m, H5-H10), 0.87 (3 H, m, H12).

2-Dec-9'-enyl-2-oxazoline Dc$^=$Ox was synthesized from 91.2 g (0.495 mol, 1 eqv.) of undec-10'-enoic acid, 45.3 g of ethanolamine (0.742 mol, 1.5 eqv.) and 0.8 g (2.4 mmol) of titanium(IV) butoxide, which were added without any additional solvent in a flask and were stirred under reflux at 130 °C for 24 h. Further preparation and purification were performed analogously to the synthesis of NonOx. 62 g (0.296 mol) of purified product were obtained; yield: 60%.

^{1}H-NMR (300 MHz, $CDCl_3$) ; for atom numbering, see Scheme 1: δ (ppm) = 5.90-5.70 (1 H, m, H12), 5.00-4.86 (2 H, m, H13), 4.19 (2 H, t, $^3J_{H,H}$ = 9.3 Hz, H2), 3.79 (2 H, t, $^3J_{H,H}$ = 9.3 Hz, H1), 2.24 (2 H, m, H4), 2.06-1.96 (2 H, m, H11), 1.68-1.52 (2.02, 2 H, m, H5), 1.40-1.20 (10 H, m, H6-H10).

Synthesis of pNonOx$_{80}$-*stat*-pDc$^=$Ox$_{20}$

For the small-scale polymerization, 3 g (15.2 mmol; 80 eqv.) of NonOx, 800 mg (3.8 mmol; 20 eqv.) of Dc$^=$Ox, 35 mg (0.19 mmol; 1 eqv.) of methyl tosylate and 6.6 g of HMIM BF_4 were added in a glass vial dedicatedly designed for operation in the Monowave 300 reactor by Anton Paar (glass type: G30). After stirring and bubbling with nitrogen, the capped vial was placed in the microwave cavity and operated at the conditions described above. For the large-scale synthesis, 216 g (1.10 mol; 80 eqv.) of NonOx, 57.6 g of Dc$^=$Ox (0.28 mol; 20 eqv.), 2.54 g (13.6 mmol; 1 eqv.) of methyl tosylate and 475 g of HMIM BF_4 were thoroughly mixed and placed in the reactor of the Anton Paar Masterwave BTR microwave reactor. The reaction mixture was operated at the conditions described above. After cooling, the copoly(2-oxazoline)

precipitated and could be filtered off the IL. The polymer contained residual amounts of IL, which could be isolated from the polymer by ultrasonic treatment in water; the recovered IL was dried and added to the HMIM BF_4 recovered by filtration. The total recovery rates were found to be 455 g (95.7%) for the IL HMIM BF_4 and 261 g (95.4%) for the copolymer pNonOx$_{80}$-*stat*-pDc$^=$Ox$_{20}$.

^{1}H-NMR (300 MHz, $CDCl_3$); for atom numbering, see Scheme 2: δ (ppm) = 5.81 (20 H, m, H12'), 4.94 (40 H, m, H13'), 3.43 (400 H, m, H1, H2 and H1', H2'), 2.23 (200 H, m, H4 and H4'), 2.02 (40 H, m, H5'), 1.61 (160 H, m, H5), 1.26 (1200 H, m, H6-H11 and H6'-H11'), 0.89 (240 H, m, H12).

IR: ν (cm$^{-1)}$ = 2921 *m*, 2852 *m*, 1641 *s*, 1463 *m*, 1430 *m*, 1182 *m*, 1160 *m*, 909 *w*, 772 *w*, 721 *w*.

Photoresist formulation and application

Photoresist formulations were prepared from 500 mg of pNonOx$_{80}$-*stat*-pDc$^=$Ox$_{20}$ (0.5 mmol of Dc$^=$Ox), 2.5 g of ethyl lactate, 60 mg of 4SH (0.5 mmol of SH groups) and 10 µL of the photoinitiator Lucirin TPO-L, or stoichiometric multiples thereof. The formulation was spincast onto FR4 substrates, gold-coated FR4 plates, glass plates or calcium fluoride plates (1 mL per 25 cm^2), respectively, at 2500 rpm during 30 s, yielding polymer films with a height of 100 nm according to Dektak measurements. After drying at 80 °C for 3 min, the film was illuminated through a mask with a geometric pattern. The resist was subsequently developed in ethyl lactate at elevated temperatures.
